# Delayed presentation of a retained colonic segment in a child with intestinal failure on teduglutide

**DOI:** 10.1002/jpr3.70049

**Published:** 2025-06-19

**Authors:** Rachel C. Bordelon, Sarah J. Varalla, Varaha S. Tammisetti, Mohamed M. Shahin, Amanda Tchakarov, Essam Imseis, Allison L. Speer

**Affiliations:** ^1^ Department of Pediatric Surgery McGovern Medical School at the University of Texas Health Science Center at Houston Houston Texas USA; ^2^ Short Bowel Syndrome Therapy and Rehabilitation (STAR) Team, McGovern Medical School, The University of Texas Health Science Center at Houston and Children's Memorial Hermann Hospital Texas Medical Center Houston Texas USA; ^3^ Department of Diagnostic and Interventional Imaging McGovern Medical School at the University of Texas Health Science Center at Houston Houston Texas USA; ^4^ Department of Pathology McGovern Medical School at the University of Texas Health Science Center at Houston Houston Texas USA; ^5^ Division of Pediatric Gastroenterology, Hepatology, and Nutrition, Department of Pediatrics McGovern Medical School at the University of Texas Health Science Center at Houston Houston Texas USA

**Keywords:** enteral autonomy, intestinal adaptation, laparotomy, nutrition, short bowel syndrome

## Abstract

Teduglutide is a glucagon‐like peptide 2 (GLP‐2) analogue that was approved by the United States Food and Drug Administration for the treatment of pediatric (>1 year) intestinal failure due to short bowel syndrome in 2019. GLP‐2 analogues promote rapid intestinal adaptation, increasing the absorptive capacity of residual intestine after surgical resection to aid the achievement of enteral autonomy or reduce parenteral nutrition requirements. Despite relatively few reported side effects, there is a theoretical risk of proliferative complications. Here we present an intriguing case of a pediatric patient found to have a decade‐long retained, discontinuous segment of colon from a surgical procedure performed in infancy, which became clinically significant after a period of treatment with teduglutide.

## 1 INTRODUCTION

Pediatric intestinal failure (PIF) is caused by reduction of functional intestine below the threshold to support growth and development, often the result of short bowel syndrome (SBS) after surgical resection.^1^, ^2^ Outcomes have improved with implementation of parenteral nutrition (PN) and multidisciplinary intestinal rehabilitation teams, but morbidity remains significant.^3^, ^4^

PIF management includes maximizing factors which promote enteral autonomy from PN. Teduglutide, a degradation‐resistant glucagon‐like peptide 2 (GLP‐2) analog, has been used in the treatment of PIF since 2019. GLP‐2 stimulates crypt cell proliferation, inhibits enterocyte apoptosis, and increases intestinal blood flow.^5^ In patients with PIF, teduglutide likely increases absorptive surface area via mucosal expansion and villus lengthening, accelerating intestinal adaptation to promote enteral autonomy or reduce PN requirements.^6^ Few adverse effects have been reported, but there is a theoretical risk of growth‐related complications. Though it has not been in use long enough to analyze long‐term risks in pediatric patients, there is currently no conclusive evidence that teduglutide promotes intestinal polyp formation or neoplasia in adults.^7^ Here we discuss a pediatric patient found to have a discontinuous colonic segment, which may have grown after treatment with teduglutide.

## 2 CASE REPORT

A twin male was born at 30 weeks gestation at a county hospital with a Level III neonatal intensive care unit. On day of life 11, he developed necrotizing enterocolitis and underwent resection of 90% of his small bowel, ileocecectomy, and partial right colectomy. An end jejunostomy was created. The distal colon remained in situ with the proximal end near the hepatic flexure. He had 17 cm of jejunum remaining (16% of his expected small bowel length‐for‐age^8^) and required long‐term PN. At 10 months of age, his jejunostomy was reversed at the same county hospital. Identification of the proximal end of the colon was challenging due to dense adhesions in the right upper quadrant. An on‐table contrast enema with fluoroscopy identified what was thought to be the end of the transverse colon. This was mobilized and transected, and an end‐to‐end jejunocolonic anastomosis was completed. His postoperative course was uneventful. He received enteral nutrition with supplemental PN managed by our intestinal rehabilitation team. At 7 years of age, he began teduglutide treatment. At the time, PN provided 25% of his energy needs, and his enteral nutrition (oral and continuous gastrostomy tube feeds), though high in calories, was largely malabsorbed due to SBS. Within 1 year of starting teduglutide, he weaned off PN, maintained a healthy weight, and achieved enteral autonomy.

**Figure 1:**
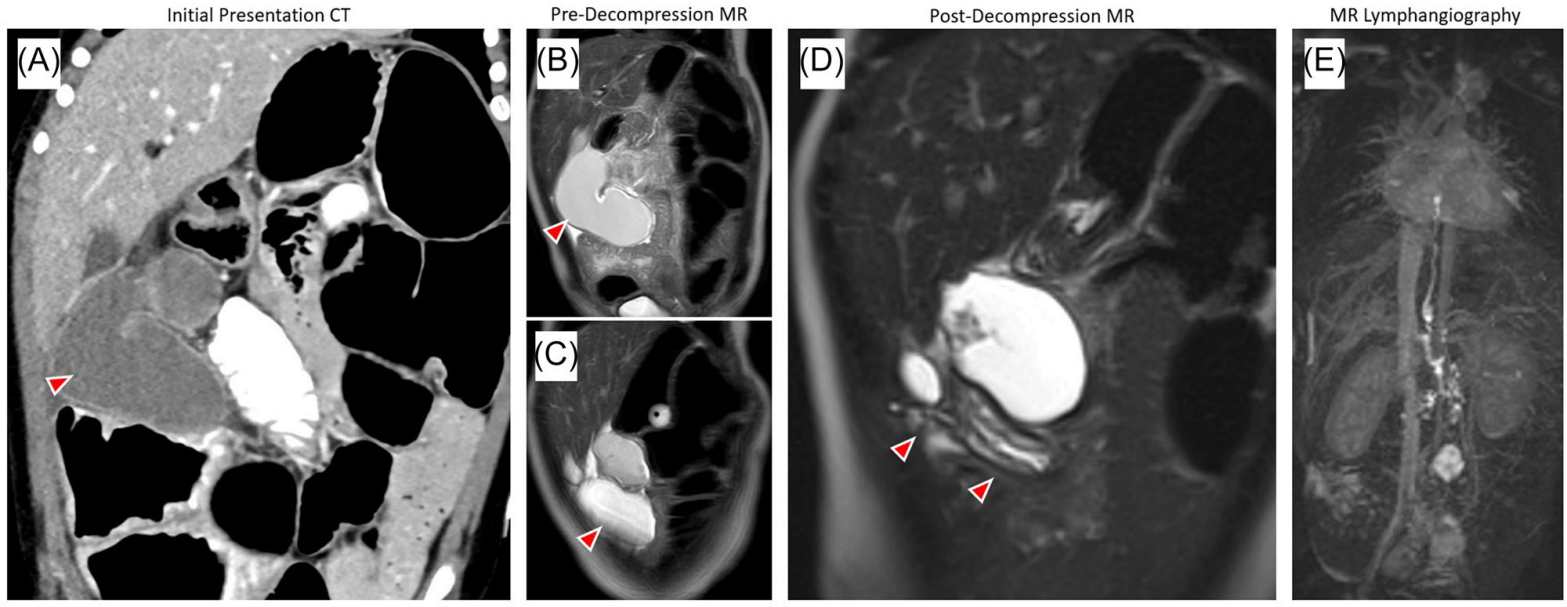
(A) Coronal computed tomography (CT) of the abdomen and pelvis with intravenous and oral contrast showing two fluid collections in the right abdomen; the lateral collection (arrow) is elongated and tubular. (B, C) Coronal T2‐weighted (T2W) magnetic resonance (MR) showing the fluid collections. (D) Coronal T2W MR as part of Dynamic MR Lymphangiogram (MRL) with a drainage catheter decompressing the lateral tubular collection (arrows), which showed mural stratification resembling gut layers (hypointense inner‐layer/mucosa, hyperintense mid‐layer/submucosa, hypointense external layer/muscularis, and serosa). Mural stratification only appeared after decompression. (E) Coronal T1‐maximum intensity Dynamic MRL image showing contrast in retroperitoneal lymphatic ducts and thoracic duct; no communication or leak observed.

At 10 years of age, he developed abdominal pain and nausea and presented to a different pediatric hospital. Computed tomography (CT) revealed two right upper quadrant fluid collections, the larger over 10 cm (Figure 1A–C). Magnetic resonance imaging confirmed a tubular, thick‐walled collection with nonspecific internal contents and an adjacent smaller collection. After review with multiple radiologists, these were considered most consistent with complicated lymphoceles. A CT‐guided percutaneous drain was placed to decompress the larger collection (Figure 1D). The effluent was clear and viscous; cultures were negative. Sclerotherapy was performed, his symptoms improved, and the drain was removed before discharge. However, he returned 2 weeks later with similar symptoms. Ultrasound showed reaccumulation of a large, unilocular fluid collection. Another percutaneous drain was placed for symptom management. Cytology showed inflammatory debris; repeat cultures were negative.

**Figure 2:**
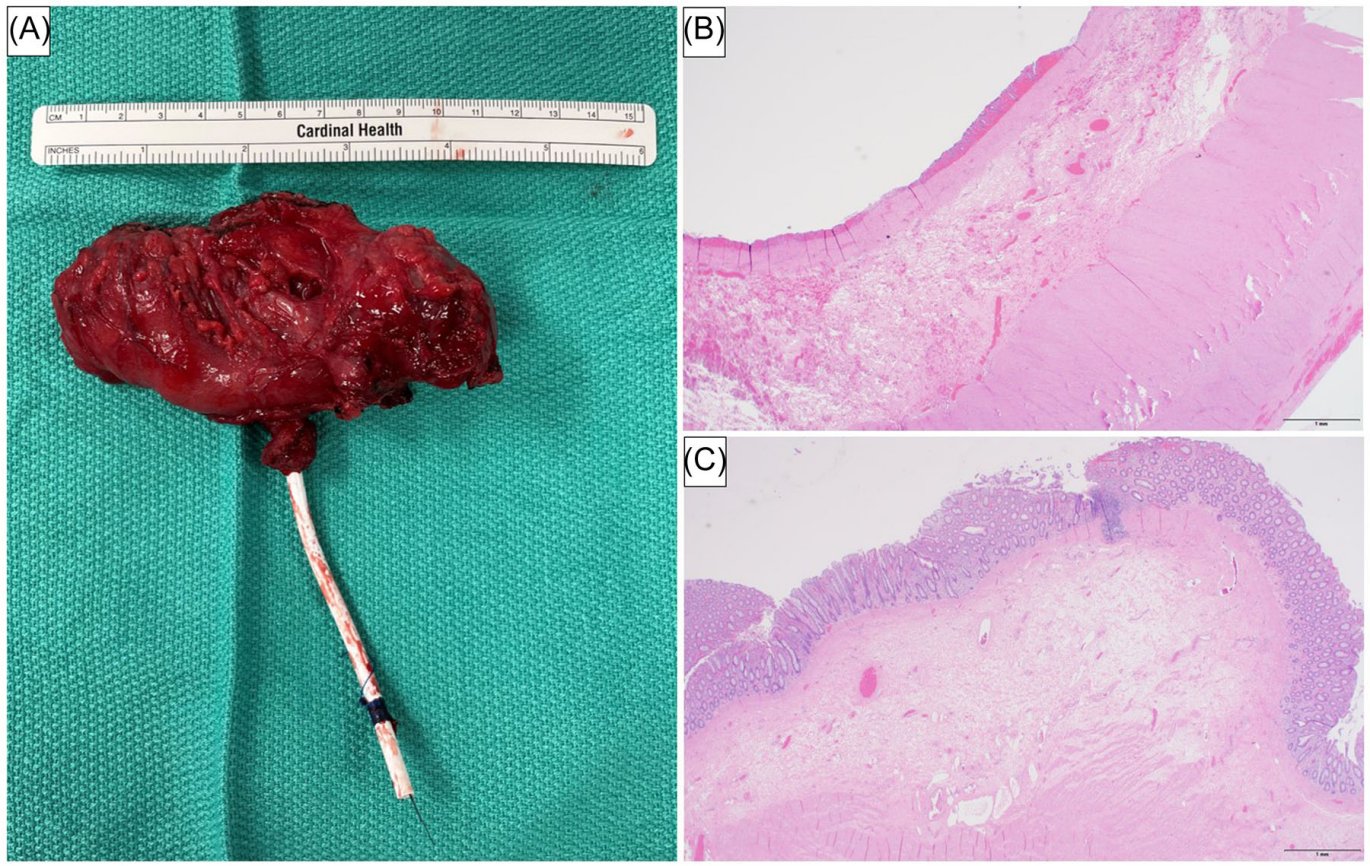
(A) Resected specimen including the percutaneous drain placed by interventional radiology. (B, C) Histologic sections revealing bowel wall with muscularis propria, submucosa, and areas of mucosal erosion (B), additionally present with sections lined by colonic‐type mucosa (C). (hematoxylin and eosin staining, 20x magnification, scale bar = 1 mm).

Multidisciplinary discussion and review of the county hospital's operative records led our team to suspect that the fluid collection was actually a retained segment of the colon at the hepatic flexure. Lymphangiography confirmed no discernible connection between the collection and the lymphatic system (Figure 1E). He was taken for exploratory laparotomy. After extensive adhesiolysis, the specimen was identified and resected; pathology confirmed colonic origin (Figure 2A–C).

## 3 DISCUSSION

Children with PIF from SBS often undergo multiple intestinal surgeries. These may include bowel resections, adhesiolysis, ostomy revision or reversal, and bowel lengthening. When procedures are performed by different surgeons at different hospitals, ensuring an accurate understanding of a patient's unique anatomy can be difficult. This patient had an unknown discontinuous colonic segment that posed a diagnostic challenge when, years later, he developed symptoms secondary to mass effect.

**Figure 3:**
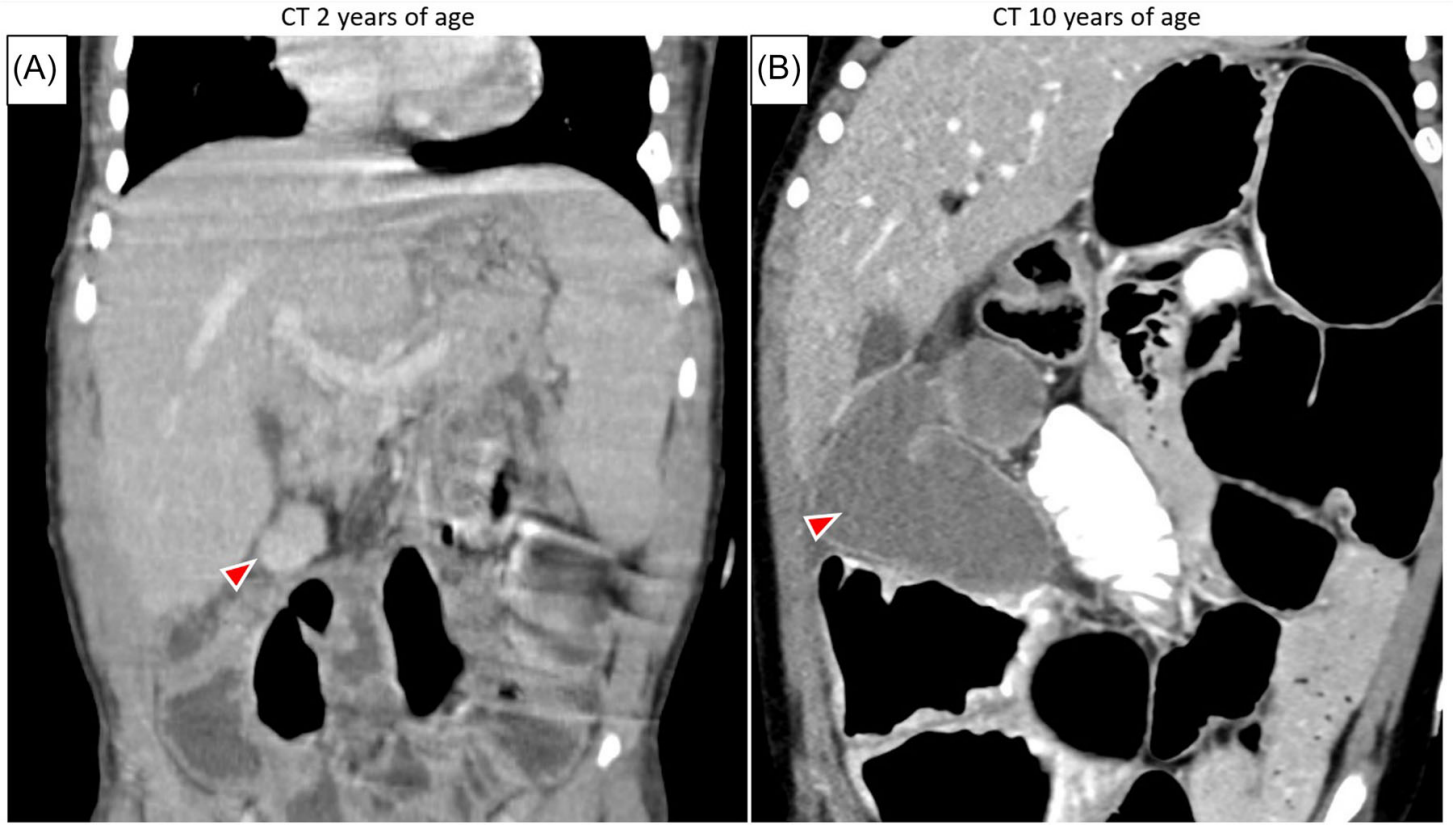
Coronal computed tomography (CT) of the abdomen and pelvis with intravenous (A, B) and oral (B) contrast showing significant growth of the excluded colonic segment over time. Though not detected at the time, the discontinuous segment (arrow) can be visualized in the right upper quadrant of a scan obtained when the patient was 2 years of age (A), 8 years before the events and imaging described in this report (B). Of note, the CT at 2 years of age was obtained during workup for sepsis and revealed an abdominal wall abscess near his gastrostomy tube.

It is difficult to determine whether teduglutide played a causative role in this complication. While it is possible that intestinal adaptation augmented by the proliferative effects of teduglutide accelerated growth of the colonic segment, it became clinically apparent 3 years after initiation of therapy, and the only prior cross‐sectional imaging available for comparison was obtained when the patient was 2 years old. In retrospect, the colonic segment is visible on that study but was very small and not detected at the time (Figure 3). Nonetheless, as the use of teduglutide is relatively new in the management of PIF, we believe it is important to consider this as a potential adverse effect.

This case highlights several key points. Children with PIF often have complex surgical histories and unique gastrointestinal anatomy. Meticulous procedural documentation and careful review of previous records are imperative in the surgical management of these patients. Our patient required extensive workup and multiple procedures before the correct diagnosis was made, which potentially could have been avoided with a clearer surgical history. From an operative perspective, leaving bowel in discontinuity for long periods of time should be avoided to minimize complications as well as confusion and misdiagnosis in the future. In circumstances where it is absolutely necessary to leave discontinuous intestine in situ, this should be clearly documented in the operative note and explained to the patient and their caregivers. Regarding nutrition management, it is remarkable that this child was able to achieve enteral autonomy given the severity of his SBS, emphasizing the benefit of GLP‐2 analogues in the treatment of PIF. Finally, though it is inconclusive whether this was truly a proliferative complication of teduglutide, our experience suggests it is wise to resect any retained, blind segment of bowel before starting teduglutide.

## 4 CONCLUSION

This case suggests exercising caution when initiating teduglutide in children with discontinuous bowel, as there may be potential for growth‐related complications.

## CONFLICT OF INTEREST STATEMENT

The authors declare no conflicts of interest.

## ETHICS STATEMENT

Informed parental consent was obtained for publication of case details and selected imaging.
